# ABCG2-overexpressing H460/MX20 cell xenografts in athymic nude mice maintained original biochemical and cytological characteristics

**DOI:** 10.1038/srep40064

**Published:** 2017-01-06

**Authors:** Wei Zhang, Zhen Chen, Likun Chen, Fang Wang, Furong Li, Xiaokun Wang, Liwu Fu

**Affiliations:** 1 Experimental Animal Center, Sun Yat-sen University, Guangzhou, 510080, China; 2 Collaborative Innovation Center for Cancer Medicine, State Key Laboratory of Oncology in South China, Sun Yat-Sen University Cancer Center, Guangzhou 510060, China; 3 Guangdong Esophageal Cancer Institute, Guangzhou, 510060, China

## Abstract

H460/MX20 are derived from large cell lung cancer H460 cell line and then transformed into ABCG2-overexpressing cells by mitoxantrone’s induction, which are widely used in study of multidrug resistance (MDR) *in vitro*. To establish and spread the model of H460/MX20 cell xenografts, we investigated whether cell biological characteristics and the MDR phenotype were maintained *in vivo* model. Our results demonstrated that the cell proliferation, cell cycle, and ABCG2 expression level in xH460/MX20 cells isolated from H460/MX20 cell xenografts were similar to H460/MX20 cells *in vitro*. Importantly, xH460/MX20 cells exhibited high levels of resistance to ABCG2 substrates such as mitoxantrone and topotecan as H460/MX20 cells did. Furthermore, lapatinib, the inhibitor of ABCG2, potently reversed mitoxantrone- and topotecan-resistance of xH460/MX20 cells. Taken together, these results suggest that H460/MX20 cell xenografts in athymic nude mice still retain their original cytological characteristics and MDR phenotype. Thus, the H460/MX20 cell xenografts model could serve as a sound model *in vivo* for study on reversal MDR.

Chemotherapy is the main means of cancer treatment, but it is impeded with the development of resistance to multiple chemotherapeutic drugs^1^. Among the existing mechanisms, the major and most well-studied form is multidrug resistance (MDR). MDR is mainly due to the overexpression and regulation of the transmembrane ATP-binding cassette (ABC) transporters that function as active drug efflux pumps. They pump anti-cancer agents from intracellular to extracellular space, causing resistance of tumor cells. Hitherto, 49 different ABC transporter family members have been identified in the human genome and divided into 7 distinct subfamilies (designated A to G) on the basis of similarities in sequence and structural organization^2^. Thereinto, ABC subfamily G member 2 (ABCG2; also called breast cancer resistance protein, BCRP) is an important members of ABC transporters that have been involved in the development of MDR in tumor cells^1^,^3^,^4^.

Mounting evidence showed that the overexpression of ABCG2 was positively correlated with a poor response to chemotherapy in clinical practice^5^,^6^,^7^,^8^. Doyle *et al*. discovered that ABCG2 was overexpressed in MCF/AdrVp cells compared to parental MCF-7 cells using RNA fingerprinting^9^. ABCG2 can translocate some chemotherapeutic drugs across cellular membranes, including mitoxantrone, topotecan, methotrexate and flavopiridols and *etc*^10^,^11^. It has been reported that chemotherapeutic efficacy in patients of non-small cell lung cancer is positively correlated with the level of ABCG2 expression and independent on ABCB1, ABCC1 and ABCC3, suggesting that specific suppression of ABCG2 may help to circumvent MDR in these patients^12^.

Although the best-known role for ABCG2 is the ability to protect tumor cells from death by effluxing anticancer drugs from target cells. Its overexpression was later reported to be associated with radiation resistance and worse clinical outcome in both lung cancer^13^ and medulloblastoma^14^. Besides, ABCG2 could protect normal cells, tumor and stem cells from apoptosis in the presence of stress of non-ABCG2 substrates^15^,^16^,^17^. Human embryonic stem cells can better tolerate physical damage, drugs and UV light with the presence of ABCG2^18^. In normal placenta, knockdown of ABCG2 by siRNA or inhibition of ABCG2 significantly restored the sensitivity of placental trophoblasts to apoptotic stress in response to cytokines^16^. These studies strongly indicate that ABCG2 could play a key role in survival, which is much broader than its currently established role in drug efflux^19^. So establishing an ABCG2 overexpressing *in vivo* model would be effective and necessary for illustrating the mechanism of MDR, as well as giving support to further study the anti-apoptosis ability of specific cells.

H460/MX20 derived from large cell lung cancer H460 cell line was mitoxantrone-induced ABCG2-overexpressing cells that have been widely applied to ABCG2-mediated MDR research including xenografts model^20^,^21^. However, according to the related reports, MCF-7/Adr cells were cultured in the absence of doxorubicin at 4- to 5-week intervals and their sensitivity to doxorubicin increased in a time-dependent manner^22^. *In vitro*, H460/MX20 was also cultured additionnally in the mitoxantrone (20 nM) to maintain ABCG2 expression^23^. Though H460/MX20 cell xenograft had been used in some reports, most of these research was based on the hypothesis that H460/MX20 can maintain its resistance *in vivo*. However, there were no direct evidences to support it. Whether H460/MX20 cells in xenografts in absence of mitoxantrone would lose biological characteristics and the MDR phenotype in a time-dependent manner is still unclear. Therefore, we established H460/MX20 cell xenografts model in athymic nude mice and investigated the biological characteristics and the MDR phenotype of xenografts cells.

## Results

### The establishment of H460 and H460/MX20 cell xenografts

**Figure 1:**
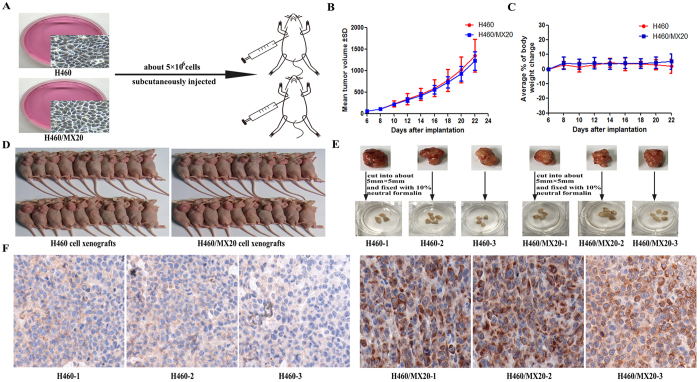
The establishment of H460 and H460/MX20 cell xenografts. (**A**) A total of 40 mice were subcutaneously inoculated with H460 and H460/MX20 cells (≈5 × 10^6^) in the right flank, respectively. (**B**) The changes in tumor volume and body weight over time following the implantation. Data points represented the mean ± SD of tumor volumes and body weight from each group. n = 20. (**C**) Solid tumor formation rate of H460 and H460/MX20 cells (100%). (**D**) The selected cell xenografts were cut into about 5 mm × 5 mm and fixed with 10% neutral formalin. (**E**) ABCG2 expression analysis by immunohistochemistry in tumor tissues collected from H460 and H460/MX20 cell xenografts.

H460 and H460/MX20 cells suspended at 5 × 10^6^ cells (200 μl) in DMEM medium were subcutaneously injected into the right flank of athymic nude mice (n = 20 per group) (Fig. 1A). The overall tumor formation rate was all 100% (20/20) (Fig. 1D). Solid tumors were measured 6 days after inoculation. The curve of tumor growth was drawn according to tumor volume and time of implantation (Fig. 1B), and there was no death and visible weight loss in these mice (Fig. 1C). At 22 days after inoculation, when the mean tumor weight was over 1 g, the mice were anesthetized and sacrificed. Then, we selected H460 and H460/MX20 cell xenografts to cut into about 5 mm × 5 mm, and fixed with 10% neutral formalin overnight (Fig. 1E). The expression of ABCG2 *in vivo* by performing immunohistochemical staining was tested, which showed high expression of ABCG2 and mainly located specifically in the cell membrane of H460/MX20, while little expression of ABCG2 in H460 cell xenografts was shown (Fig. 1F).

### The expression of ABCG2 in H460/MX20 and xH460/MX20 cells

**Figure 2:**
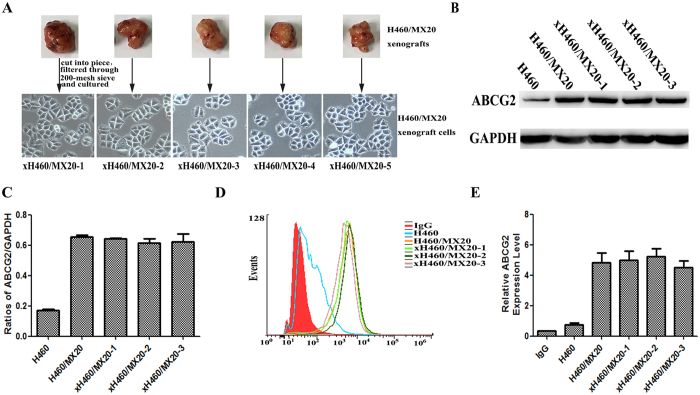
The expression of ABCG2 in H460/MX20 and xH460/MX20 cells. (**A**) The isolation and culture of xenograft cells (xH460/MX20). (**B,C**) ABCG2 protein level was analyzed by western blotting with anti-ABCG2 and statistical analysis of western blotting assay. The gray value was calculated by Image J and was normalized to the GAPDH control. (**D,E**) The cell surface expression of ABCG2 was measured by flow cytometry. The values shown were obtained from three independent experiments and were normalized to the GAPDH control.

To figure out whether the expression level of ABCG2 was changed in xH460/MX20 cell xenografts, firstly, we isolated xenograft cells from H460/MX20 tumor xenografts as described in the Materials and Methods section, named xH460/MX20 cells (Fig. 2A). Then, we examined the H460/MX20 and xH460/MX20 for ABCG2 expression. Western blotting of cell extracts with anti-ABCG2 antibody revealed that no marked difference in ABCG2 protein level in H460/MX20 and xH460/MX20 cells (Fig. 2B and C) existed. To further investigate the cell-surface expression of ABCG2 in these cell lines, flow cytometry analysis with intact cells showed that the expression of ABCG2 was almost the same level in H460/MX20 and xH460/MX20 cells (Fig. 2D and E). Taken together, these results suggested that xenograft cells could keep original biochemical properties in protein level and cell-surface expression of ABCG2.

### Proliferation characteristics of H460/MX20 and xH460/MX20 cells *in vitro*

**Figure 3:**
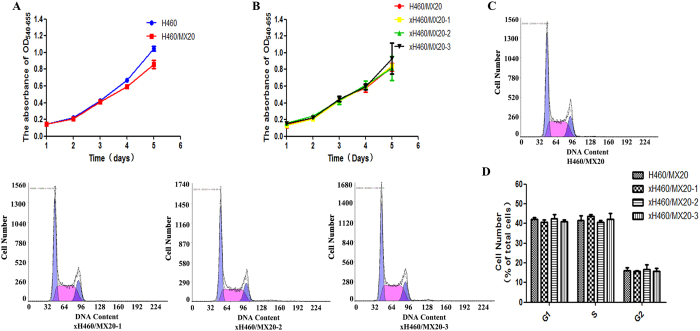
The proliferation and cell cycle analysis of H460/MX20 and xH460/MX20 cells. (**A**) The proliferation curves of H460 and H460/MX20 cells. (**B**) The proliferation curves of H460/MX20 and xH460/MX20 cells. (**C,D**) The percentages of the cell population at the G1, S, and G2 phases were calculated from histograms using the CellQuest software. Cell cycle analysis was performed on a flow cytometer. All results were presented the means ± S.D. of three independent experiments.

Growth and division properties of H460 versus H460/MX20 cells and H460/MX20 versus xH460/MX20 cells cultured in 96-well microtiter plates were compared. The proliferating ability of these cells did not change significantly in above two pairs, respectively (Fig. 3A and B). Flow cytometry with propidium iodide staining showed no statistical difference between the above two cell lines in the G1 (42.13 ± 0.84 for H460/MX20 vs. 40.57 ± 1.18 for xH460/MX20–1 or 42.47 ± 2.26 for xH460/MX20-2 or 40.87 ± 1.10 for xH460/MX20-3), S (41.7 ± 2.25 vs. 43.63 ± 0.84 or 40.77 ± 0.96 or 42.3 ± 3.04), G2 (16.2 ± 1.39 vs. 15.8 ± 0.36 or 16.7 ± 2.22 or 15.8 ± 1.44), (p > 0.05) (Fig. 3C and D).

### Cell sensitivity to chemotherapeutic agents

The drug toxicities of H460, H460/MX20, xH460 (from H460 xenograft model) and xH460/MX20 (from H460/MX20 xenograft model) measured by MTT assays were shown in Table 1. H460/MX20 cells were 37-fold more resistant to mitoxantrone than parental H460 cells and displayed 142-fold resistance to topotecan. Similarly, xH460/MX20 cells were 33.7-fold more resistant to mitoxantrone than parental xH460 cells and displayed 141.5-fold resistance to topotecan. Lapatinib (1.25 μM), the inhibitor of ABCG2, effectively reversed mitoxantrone and topotecan resistance in H460/MX20 cells (fold-reversal was 11.3 and 65.4, respectively). To these agents, similar results were also obtained with xH460/MX20 cells (fold-reversal was 10.4 and 67.1, respectively). There was no reversal effect in H460 and xH460. These results indicated that xH460/MX20 still maintained its resistance, while xH460 remained sensitive.

### Intracellular accumulation of Hoechst 33342

**Figure 4:**
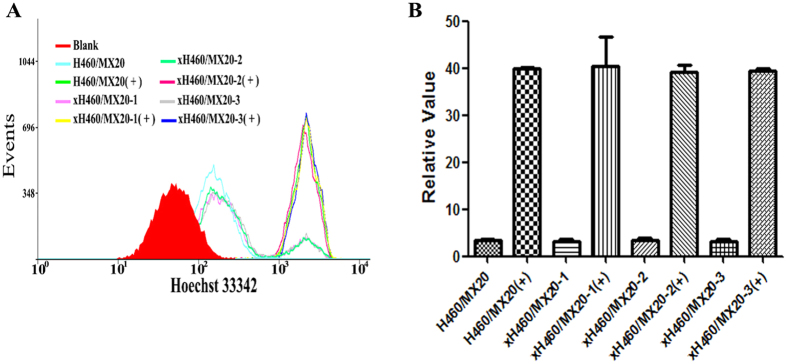
The intracellular accumulation of Hoechst 33342 in H460/MX20 and xH460/MX20 cells. The intracellular accumulation of Hoechst 33342 was measured by flow cytometry. +: the sample was treated with lapatinib (1.25 μM). Data were represented the mean ± SD of at least three independent experiments. Events represented the number of cells at different fluorescence intensities.

The intracellular accumulation of Hoechst 33342 was 0.98 or 1.01 or 0.99 fold higher, respectively, in H460/MX20 than in xH460/MX20-1 or xH460/MX20-2 or xH460/MX20-3 cells. In the presence of lapatinib (1.25 μM), the level of Hoechst 33342 accumulation was enhanced by 12.2- and 12.5- or 11.9- or 12.3-fold for H460/MX20 and xH460/MX20-1 or xH460/MX20-2 or xH460/MX20-3 cells, respectively (Fig. 4A and B). These results were indicative of similar ABCG2 transport activity of these cell lines.

## Discussion

Athymic nude mice models of tumor cells have been established to better study the molecular mechanisms of the occurrence and development of cancer *in vivo*. Compared with cellular studies *in vitro*, animal models enable to further understand the precise regulatory mechanisms of MDR *in vivo* and possess promising clinical values due to the high homology between human and mice. In addition, it had been suggested that ABCG2 may also play a more general role in cell survival. Research *in vitro* existed relation in clinical circumstances, where overexpression and activity of ABCG2 was associated with radiotherapy resistance^13^. Another study reported that human embryonic stem cells expressing ABCG2 could tolerate the physical stress and UV irradiation much better than the ABCG2-negative cells^18^. Interestingly, several studies proved that ABCG2 may also serve as an indicator of poor prognosis in certain tumors, including lung^24^, breast^25^, and esophageal cancers^26^. In brief, these studies clearly stated the key role for ABCG2 in general tumor cell survival independent of its well-characterized drug efflux function. ABCG2-overexpressing H460/MX20 xenograft model may also play some role in clarifying the above mechanism.

H460/MX20 cell line which serve as a drug-resistant model with overexpression of ABCG2 has been widely applied to the research of ABCG2-mediated MDR *in vitro*. Though H460/MX20 cell xenograft had been used to investigate reversal mechanisms of telatinib and afatinib^20^,^21^, a problem existed on whether this cell xenograft was feasible or just a conceptual model? Based on previous reports and our success with other tumor xenografts^27^,^28^, we believed that the H460/MX20 cell xenografts could act as a sound model to study the functional mechanism of ABCG2-mediated MDR. And we aim to prove that this xenograft model can maintained the original cytological characteristics of H460/MX20 cells. In previous studies, the tumor cells retained their original cytological characteristics after S1-M1-80 xenograft model was established in mice^27^. The results of another Mg63/DOX cells xenograft model established indicated that the molecular features and MDR phenotype of xenograft cells were not significantly different from those of MG63/DOX cells^29^. These studies suggested that cancer cells from *in vivo* models might still maintain original biochemical and cytological characteristics. Similarly, our results showed that H460/MX20 cell xenograft in athymic nude mice could keep their biological characteristics after 22 days, which indicated that the xenograft model may be useful in preclinical studies of drug resistance, discovering novel therapeutic strategies and evaluating the efficacy of cancer treatment.

To the best of our knowledge, MDR of cancer cells is a major cause of failure in chemotherapy. It mainly manifests as reduction of intracellular accumulation of anticancer agents due to energy-dependent efflux from intracellular to extracellular spaces. It is generally known that ABCG2-overexpressing H460/MX20 cells possess obvious resistance to certain anticancer drugs, such as mitoxantrone and topotecan. But, whether the drug-resistance would occur in xH460/MX20 cells was unknown. In our experiment, H460/MX20 and xH460/MX20 cells were assayed for sensitivity to mitoxantrone and topotecan. And our data clearly showed that the drug-resistance was almost identical for H460/MX20 and xH460/MX20 cells, both having a high level of resistance to mitoxantrone and topotecan and showing no cross-resistance to cisplatin, a non-ABCG2 substrate.

The present study demonstrated that xH460/MX20 cells possessed similar cytological and biochemical characteristics as H460/MX20 cells did in the following aspects. First, compared with H460/MX20 cells, the proliferating ability and cell cycle of xH460/MX20 cells did not change significantly. Secondly, there was no obvious difference in the ABCG2 expression level between H460/MX20 and xH460/MX20 cells. Accordingly, immunohistochemical analysis showed high expression of ABCG2 in H460/MX20 cell xenograft, while very low expression in H460 cell xenograft. Thirdly, the two cell lines possessed similar level of resistance to mitoxantrone and other ABCG2 substrates, and lapatinib can further reverse MDR effectively by inhibiting the activity of ABCG2. Moreover, H460/MX20 and xH460/MX20 cells were equally sensitive to cisplatin, a non-ABCG2 substrate.

In conclusion, we have established xenograft model of H460/MX20 cells that still maintained their original biochemical and cytological characteristics *in vivo* model. Thus, athymic nude mice model of large cell lung cancer can serve as a pharmacologic model for screening new potential anticancer agents. It could be also used to study ABCG2-mediated MDR *in vivo* and other functions of ABCG2.

## Methods and Materials

### Chemicals and reagents

3-(4,5-dimethylthiazol-yl)-2,5-diphenyl-tetrazolium bromide (MTT), mitoxantrone, topotecan, cisplatin, dimethyl sulfoxide (DMSO). The ABCG2 monoclonal antibodies were purchased from Santa Cruz Biotechnology (Delaware, USA). GAPDH antibody and the secondary antibodies were purchased from Kangchen Co. (Shanghai, China). Lapatinib was purchased from LC Laboratories (Woburn, MA, USA).

### Cell lines and cell culture

Human non-small cell lung carcinoma cell line H460 and mitoxantrone-induced ABCG2-overexpressing subline H460/MX20 were generous gifts from Dr. Susan Bates (National Institutes of Health, Bethesda, MD)^30^. Cell lines were cultured in DMEM medium (Life Technologies, Carlsbad, CA, USA) supplemented with 10% fetal bovine serum and 1% antibiotic solution (penicillin-streptomycin) at 37 °C in a humidified incubator containing 5% CO_2_.

### Human large cell lung cancer xenograft models

All animal experiments were conducted in accordance with the National Guidelines for Experimental Animal Welfare (the Ministry of Science and Technology of People’s Republic Laboratory Animals 2006-09-30) at the Animal Experiment Center of Sun Yat-Sen University Cancer Center (GuangZhou, China). The animal experiments were approved by the Institutional Animal Care and Use Committee of Sun Yat-Sen University Cancer Center.

Athymic nude mice (4–6 weeks old, Center of Experimental Animals, Sun Yat-Sen University Cancer Center) were used for the H460 and H460/MX20 cell xenografts. All animals were provided with sterilized food and water. Briefly, H460 and H460/MX20 cells suspended at 2.5 × 10^7^ cells/ml in DMEM medium were subcutaneously injected into the right flank of athymic nude mice. Tumors were measured using electronic calipers every two days. Tumor size was calculated according to the following formula: tumor volume = π/6 × L × W^2^, with L and W representing the longest and the shortest tumor diameters, respectively.

### Isolation of xH460/MX20 cells

The athymic nude mice were anesthetized and sacrificed when the mean of tumor weight was over 1 g. Fresh xenografts of H460/MX20 cells separated from the mice were mechanically disaggregated with sterile crossed scalpels to obtain pieces as small as possible. After cutting, the tissue pieces was re-suspended in cell culture dish, and incubated at 37 °C overnight. Single xH460/MX20 cell suspension solution was obtained after digestion.

### Cytotoxicity determination by MTT assay

Briefly, cells were harvested and re-suspended at a final concentration of 2.5 × 10^3^ cells/well for H460, H460/MX20 and xH460/MX20. Cells were seeded evenly into (180 μl/well) 96-well plate. After incubating for 24 h at 37 °C, 10 μl of different concentrations of the anticancer drug (prepared in DMEM with final DMSO concentration of 0.1%) were added (10 μl of a fixed concentration of the inhibitor was added 1 h prior to the addition of anticancer drug). Subsequently the cells were incubated at 37 °C for 68 h. After 68 h, 20 μl MTT (5 mg/ml) was added to each well. The plates were incubated at 37 °C for 4 h. The MTT was gently aspirated from each well without disturbing the cells, and 150 μl of DMSO was added to dissolve the purple formazan crystals and then the absorbance at 540 nm (A_540_) was measured, with background subtraction at 655 nm, using the Model 550 Microplate Reader (Bio-Rad, Hercules, CA, USA). The concentrations required to inhibit cell growth by 50% (IC_50_) were calculated from survival curve using the Bliss method. The degree of resistance was calculated by dividing the IC_50_ for the MDR cells by that for the parental sensitive cells. The fold reversal of MDR was calculated by dividing the IC_50_ for cells with the anticancer drug in the absence of lapatinib by that obtained in the presence of lapatinib. All experiments were performed in triplicate.

### Cell proliferation assay and cell cycle analysis

Cells at exponential growth phase were seeded in 96-well plate (500 cells/well) in DMEM medium. Then, MTT (5 mg/ml) was added to the wells (20 μl/well) every 24 h for 5 consecutive days. The A_540_ was detected, with background subtraction at 655 nm. The cell proliferation curve was drawn according to A_540–655_ and days.

Cell ploidy was used to assess cell cycle status. Exponentially growing cells were harvested, fixed with cold 70% ethanol overnight, washed twice with 1 × PBS, and stained with propidium iodide (100 μg/ml) containing RNase A (100 μg/ml) for 30 min at 37 °C in the dark. Cell cycle was detected using flow cytometer (Beckman Coulet, Miami, FL). The percentages of the cell population at different phases were calculated from histograms using the CellQuest software (BD Sciences, San Jose, CA, USA).

### Western blot analysis

Cells at 90% confluence were washed once with PBS (PH 7.4) and harvested by scraping and centrifugation at 800 × g for 5 min. The harvested cells were washed with PBS and lysed for 45 min on ice in cell lysis buffer containing 20 mM Tris-HCl, pH 7.5, 150 mM NaCl, 10 mM NaF, 20 mM β-glycerophosphate, 1 mM sodium orthovanadate, 1 mM PMSF, 10 μg/ml leupeptin, 2 μg/ml aprotinin, 1% Triton X-100 and 1 mM EDTA. An equal amount of protein from each lysate was separated using SDS-PAGE and transferred onto PVDF membranes. These were incubated with primary antibodies and HRP-conjugated secondary antibodies, and visualised with Immobilon Western Chemiluminescent HRP Substrate (EMDMillipore) using film or a ChemiDoc (Bio-Rad, Hercules, CA, USA). Protein concentration was determined by the BCA assay (Thermo Pierce, Waltham, MA, USA).

### Detection of cell surface expression of ABCG2 by flow cytometer

H460/MX20 and xH460/MX20 cells were collected and washed three times with PBS buffer (supplemented with 0.5% bovine serum albumin). For ABCG2 expression analysis, FITC-conjugated anti-human Bcrp1/ABCG2 (Santa Cruz, CA, USA) reagent was mixed with 25 μl of Fc-blocked cells (1 × 10^6^ cells/ml). After incubating for 45 min at 4 °C, the cells were washed twice with PBS buffer (supplemented with 0.5% BSA) and re-suspended in 400 μl PBS buffer for flow cytometric analysis. Isotype control samples were treated in an identical manner with FITC-labeled mouse immunoglobin G2b (IgG2b) antibody. All experiments were repeated at least three times.

### Intracellular Hoechst 33342 accumulation assay

The intracellular accumulation assay of Hoechst 33342 was performed as described by Goodell and colleagues with some modifications^31^. In brief, cells were incubated in 6-well plates with exposure to 1.25 μM lapatinib for 3 hours. Then Hoechst 33342 dye (0.5 μg/ml) was added and further incubated for 1.5 hours. Finally, cells were washed with ice-cold PBS three times and resuspended in PBS for flow cytometry analysis.

### Immunohistochemical staining

Tumors were collected, fixed in 10% neutral buffered formalin (NBF) and embedded in paraffin. Sections were cut at 5-mm, deparaffinized by xylene and rehydrated in decreasing concentration of ethanol. Boiling sections on 10 mM citrate buffer for 20 mins achieved antigen retrieval. After blocking of endogenous peroxidase with 3% hydrogen peroxidase in methanol, non-specific reaction was blocked with 1% BSA at room temperature for 30 mins. The sections were next incubated with the primary antibody against ABCG2 at the dilution of 1:150, at 4 °C overnight. After washing in PBS for three times, sections were incubated with labeled polymer-HRP anti-mouse (DAKO) secondary antibody at room temperature for 1 h. The sections were then exposed to DAB (diaminobenzidine tetrahydrochloride) solution and counter stained with hematoxylin. Finally, sections were dehydrated in increasing concentration of ethanol, cleared in xylene and sealed with neutral balsam. Images were taken by Nikon EclipseE600 microscope using NIS Elements D3.0 software.

### Statistical analysis

All experiments were repeated at least three times, and the results were showed as mean values ± standard deviation (SD). The statistical software SPSS16.0 was used in data processing and analysis. Statistical significance was determined at *p* < 0.05 or *p* < 0.01 by the Student’s t-test.

## Additional Information

**How to cite this article**: Zhang, W. *et al*. ABCG2-overexpressing H460/MX20 cell xenografts in athymic nude mice maintained original biochemical and cytological characteristics. *Sci. Rep.* **7**, 40064; doi: 10.1038/srep40064 (2017).

**Publisher's note:** Springer Nature remains neutral with regard to jurisdictional claims in published maps and institutional affiliations.

## Figures and Tables

**Table 1:** Cytotoxicity of mitoxantrone, topotecan, and cisplatin and the reversal effectsof lapatinib in H460, H460/MX20, xH460 and xH460/MX20 cells.

Compounds	IC_50_ [μmol/L (fold-reversal)]			
	H460	H460/MX20	xH460	xH460/MX20
**Mitoxantrone**	0.0172 ± 0.0113	0.635 ± 0.38	0.0191 ± 0.0047	0.643 ± 0.167
**Mitoxantrone + Lapatinib**	0.0074 ± 0.005 (2.3)	0.056 ± 0.029 (11.3)	0.0085 ± 0.001 (2.2)	0.062 ± 0.011 (10.4)
**Topotecan**	0.039 ± 0.011	5.52 ± 1.12	0.0381 ± 0.007	5.39 ± 0.48
**Topotecan + Lapatinib**	0.0204 ± 0.0023 (1.9)	0.085 ± 0.033 (65.4)	0.0198 ± 0.013 (1.9)	0.08 ± 0.028 (67.1)
**Cisplatin**	1.47 ± 0.16	9.293 ± 1.923	1.380 ± 0.108	10.028 ± 1.919
**Cisplatin + Lapatinib**	1.56 ± 0.17 (0.9)	11.473 ± 1.184 (0.8)	1.345 ± 0.019 (1.0)	9.33 ± 1.569 (1.1)

Cell survival was measured by MTT assay as described in the Materials and Methods section. The fold reversal of MDR (given in parentheses) was calculated by dividing the IC_50_ for cells with the anticancer drug in the absence of lapatinib by that obtained in the presence of lapatinib. Data shown are obtained from three independent experiments.
