# Author Correction: ABCG2-overexpressing H460/MX20 cell xenografts in athymic nude mice maintained original biochemical and cytological characteristics

**DOI:** 10.1038/s41598-020-68768-8

**Published:** 2020-07-22

**Authors:** Wei Zhang, Zhen Chen, Likun Chen, Fang Wang, Furong Li, Xiaokun Wang, Liwu Fu

**Affiliations:** 1 Experimental Animal Center, Sun Yat-Sen University, Guangzhou, 510080 China; 2 Collaborative Innovation Center for Cancer Medicine, State Key Laboratory of Oncology in South China, Sun Yat-Sen University Cancer Center, Guangzhou, 510060 China; 3 Guangdong Esophageal Cancer Institute, Guangzhou, 510060 China

Correction to: *Scientific Reports* 10.1038/srep40064, published online 06 January 2017

This Article contains errors in Figure 1 and its accompanying legend. In Figure 1D, the nude mice image for H460/MX20 cell xenografts is a duplication of the nude mice image for H460 cell xenografts. In addition, the legend contains errors in the panel labelling. As a result,

“(**A**) A total of 40 mice were subcutaneously inoculated with H460 and H460/MX20 cells (≈5 × 10^6^) in the right flank, respectively. (**B**) The changes in tumor volume and body weight over time following the implantation. Data points represented the mean ± SD of tumor volumes and body weight from each group. n = 20. (**C**) Solid tumor formation rate of H460 and H460/MX20 cells (100%). (**D**) The selected cell xenografts were cut into about 5 mm × 5 mm and fixed with 10% neutral formalin. (**E**) ABCG2 expression analysis by immunohistochemistry in tumor tissues collected from H460 and H460/MX20 cell xenografts.”

should read:

“(**A**) A total of 40 mice were subcutaneously inoculated with H460 and H460/MX20 cells (≈ 5 × 10^6^) in the right flank, respectively. (**B, C**) The changes in tumor volume and body weight over time following the implantation. Data points represented the mean ± SD of tumor volumes and body weight from each group. n = 20. (**D**) Solid tumor formation rate of H460 and H460/MX20 cells (100%). (**E**) The selected cell xenografts were cut into about 5 mm × 5 mm and fixed with 10% neutral formalin. (**F**) ABCG2 expression analysis by immunohistochemistry in tumor tissues collected from H460 and H460/MX20 cell xenografts.”

The correct Figure 1 and its accompanying legend appear below.

**Figure 1:**
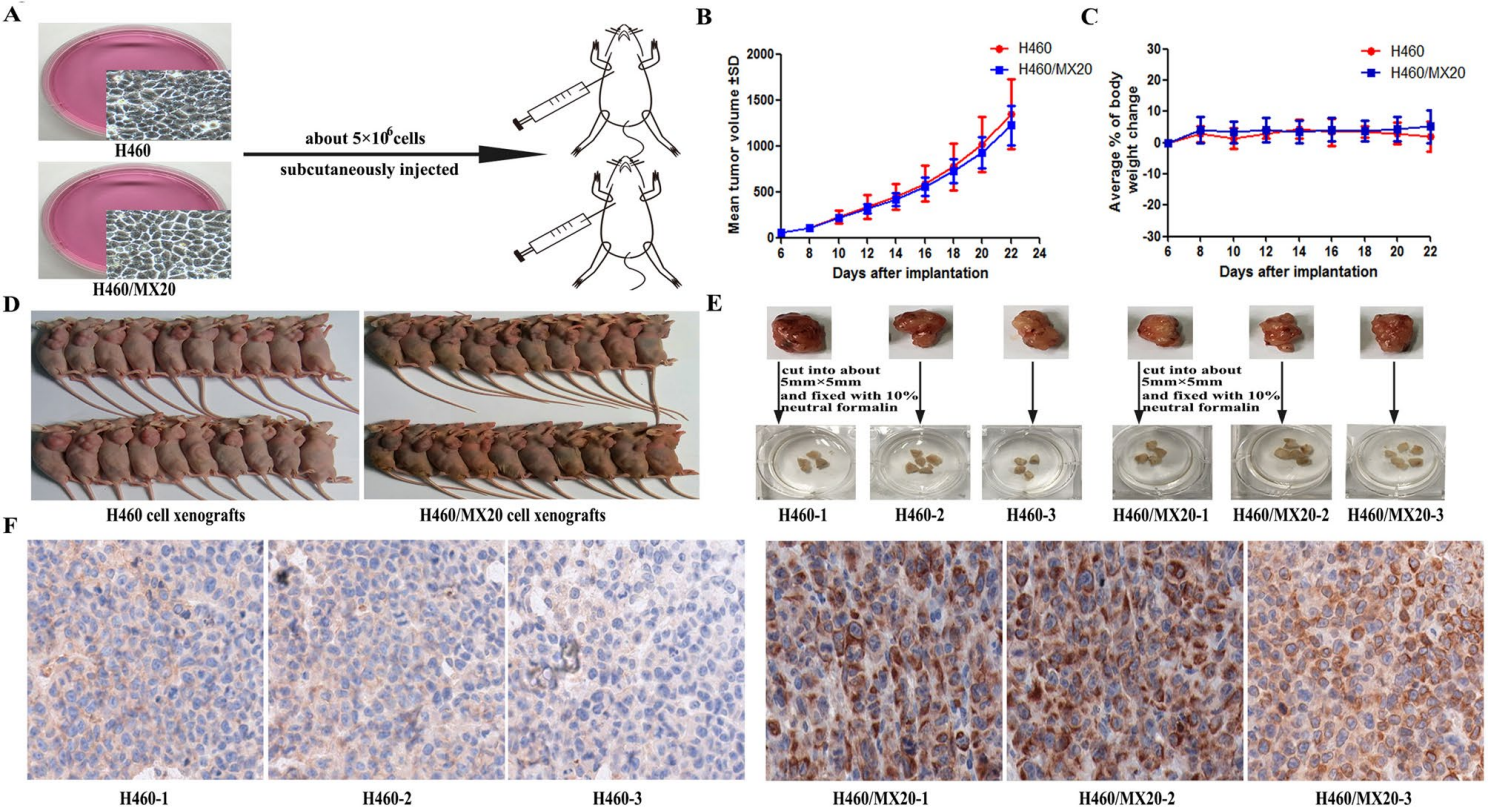
(**A**) A total of 40 mice were subcutaneously inoculated with H460 and H460/MX20 cells (≈ 5 × 10^6^) in the right flank, respectively. (**B**, **C**) The changes in tumor volume and body weight over time following the implantation. Data points represented the mean ± SD of tumor volumes and body weight from each group. n = 20. (**D**) Solid tumor formation rate of H460 and H460/MX20 cells (100%). (**E**) The selected cell xenografts were cut into about 5 mm × 5 mm and fixed with 10% neutral formalin. (**F**) ABCG2 expression analysis by immunohistochemistry in tumor tissues collected from H460 and H460/MX20 cell xenografts.

